# Evidence-Based Medicine in judicial decisions concerning right to healthcare

**DOI:** 10.1590/S1679-45082016AO3363

**Published:** 2016

**Authors:** Eduardo Rocha Dias, Geraldo Bezerra da Silva

**Affiliations:** 1 Programa de Pós-Graduação em Direito Constitucional, Universidade de Fortaleza, Fortaleza, CE, Brazil.; 2 Programa de Pós-Graduação em Saúde Coletiva, Universidade de Fortaleza, Fortaleza, CE, Brazil.

**Keywords:** Evidence-Based Medicine, Right to Health, Health/legislation & jurisprudence

## Abstract

**Objective:**

To analyze, from the examination of decisions issued by Brazilian courts, how Evidence-Based Medicine was applied and if it led to well-founded decisions, searching the best scientific knowledge.

**Methods:**

The decisions made by the Federal Courts were searched, with no time limits, at the website of the Federal Court Council, using the expression “Evidence-Based Medicine”. With regard to decisions issued by the court of the State of São Paulo, the search was done at the webpage and applying the same terms and criterion as to time. Next, a qualitative analysis of the decisions was conducted for each action, to verify if the patient/plaintiff’s situation, as well as the efficacy or inefficacy of treatments or drugs addressed in existing protocols were considered before the court granted the provision claimed by the plaintiff.

**Results:**

In less than one-third of the decisions there was an appropriate discussion about efficacy of the procedure sought in court, in comparison to other procedures available in clinical guidelines adopted by the Brazilian Unified Health System (*Sistema Único de Saúde*) or by private health insurance plans, considering the individual situation. The majority of the decisions involved private health insurance plans (n=13, 68%).

**Conclusion:**

The number of decisions that did consider scientific evidence and the peculiarities of each patient was a concern. Further discussion on Evidence-Based Medicine in judgments involving public healthcare are required.

## INTRODUCTION

Much has been discussed about judicialization of access to healthcare in Brazil. The high degree of judicial interference in the determination of provision of medications and other types of health care affects the public budgets and equity in care of its beneficiaries.^1^ In the realm of health insurance plans, judicial intervention has also been increasing sought by consumers with the intention of implementing their rights.^2^ In addition to the doctrine,^3^ the very Judiciary system itself has sought to refer the discussion of the problem, such as in the public hearing held between April and May 2009, by the Federal Supreme Court.^4^

The National Council of Justice (*Conselho Nacional de Justiça* - CNJ), an agency that controls administrative and financial activity of the Judiciary Power, issued Recommendation 31, of March 30, 2010,^5^ exhorting Federal and State Courts to enter into agreements to provide technical support by physicians and pharmacists in order to aid the judges make an opinion when considering clinical issues in health-related deeds. It also requested the courts to give orientation to the judges to avoid authorizing supply of medications not yet registered by the National Health Surveillance Agency (*Agência Nacional de Vigilância Sanitária* - ANVISA) or of investigational drugs.

Moreover, the CNJ determined the National Judiciary Forum to monitor and resolve the healthcare demands. It established the Health Forum through resolution number 107, of April 6, 2010,^6^ and later it also addressed health insurance plans.

Thus, the topic of judicialization of health recommends the adoption of solutions guided by the best techniques, which unfortunately, does not always occur. In order to orient the judicial decision as to provision of a certain treatment seeking the courts, the approach known as Evidence-Based Medicine seems to offer interesting contributions. Not by chance, the search for the best scientific knowledge, including new technologies, was accepted by the Brazilian legislation, as provided in Law number 12,401, of April 28, 2011, which altered Law number 8,080/1990, inserting Chapter VIII into its Title II. As provided in Article 19-Q of Law number 8,080/1990, added by Law number 12,401/2011, in the inclusion, exclusion, or changes by the Brazilian Unified Health System (*Sistema Único de Saúde* - SUS) of new medications, products, and procedures, as well as in the establishment or alteration of a clinical protocol or a therapeutic guideline, the scientific evidence regarding efficacy, accuracy, effectiveness and safety of the medication, product, or procedure which is the object of the process should be considered.

On the other hand, the Article 19-T of Law number 8,080/1990, prohibits the payment, compensation, or reimbursement of a medication, product, and experimental clinical or surgical procedure; or their use if not authorized by the ANVISA. In addition, dispensing, payment, compensation or reimbursement of national or imported medication and product not registered in the above-mentioned agency.

Hence, two types of solution are sought regarding judicialization: first, which is internal to the Judiciary Power, by means of the public hearing mentioned above, and by acts of the CNJ, which does not bind the judges, but establishes guidelines that can lead to better founded decisions. Second, by means of the Executive Power, by Law number 12,401/2011, in foreseeing scientific criteria for adopting new technologies, which also becomes material that the Judiciary will have to analyze when considering the requests by the parties.^7^

It is necessary to verify how the Judiciary has performed as to the requirement of scientific efficacy of the procedures and medications. By determining the provision of a medication or a treatment, has it considered the existing clinical protocols? In order to not apply the protocols, have the decisions taken into account the peculiarities of the patients and the inefficacy of the treatments made available by SUS or by private health insurance plans? Have the recommendation of the CNJ been followed?

## OBJECTIVE

To analyze, based on examination of the decisions issued by the Brazilian courts, how Evidence-Based Medicine was applied and if this led to well-founded decisions from the perspective of better scientific knowledge.

## METHODS

The study presents a critical analysis of Brazilian court decisions, three of them rendered by Federal Regional Courts and 16 issued by Court of the State of São Paulo, regarding right to health and taking into consideration Evidence-Based Medicine.

The choice of the decision of the Federal Courts resulted from a search, with no time limits, at the Federal Court Council website, dedicated to unified research of jurisprudence.^8^ This is a website where one can search the decisions issued by the Federal Supreme Court and by the Supreme Court, courts of the highest hierarchy in Brazil and responsible for the standardization and interpretation of the Constitution and laws, respectively. At this website, one can also investigate decisions rendered by the five Federal Regional Tribunals, which consider appeals of decisions in which the Union and Federal entities are stakeholders, by means of the National Jurisprudence Standardization Group (*Turma Nacional de Uniformização de Jurisprudência -* TNU), by the Regional Standardization Groups, and by the Appeals Groups of the Federation States, which also evaluate appeals of decisions made in suits that comply with the law of the Special Federal Courts, of interest to the Union and to Federal entities. The search could be made using specific terms, separated or together, by the use of modals such as “and”, “or”, “adj”, and others. The search was for decisions issued by the referred agencies of the Judiciary Power that contained the expression “Evidence-Based Medicine” (between quotes). Thus, the only decisions indicated were those in which the complete expression was mentioned. The actions at law involving the right to healthcare may involve all the entities of the Federation (Union, States, Municipalities, and Federal District). When the Union is one party, the competence for such actions belongs to Federal Court. These are decisions involving the SUS. Only three sentences (decisions of the panel of courts), of the Federal Regional Courts from the 2^nd^, 5^th^, and 4^th^ Regions, with headquarters in the cities of Rio de Janeiro (RJ), Recife (PE), and Porto Alegre (RS), respectively, were identified.

As to the sentences of the Court of the State of São Paulo, the search was made at its webpage, in the part dedicated to jurisprudence search,^9^ also by means of the expression “Evidence-Based Medicine”, and with no time limits. Similar to the Federal Court Council website, the search could be made by isolated terms or those mentioned together in the decisions. The complete expression in quotation marks was also investigated, which allowed identifying the decisions in which it had been mentioned. Since it is a State Court, the Union is not one party in these actions against private health insurance plans, and against the State of São Paulo and/or Municipalities of this Federation unit, referring to the SUS, in the second case.

The search was made at the website of the Court of the State of São Paulo, since it is the sate with the largest population and the greatest coverage by private health insurance plans, as per data of the National Agency for Private Health Insurance Plans.^10^

Nineteen Brazilian court decisions were identified, three of them issued by the Federal Regional Courts and 16 by the Court of the State of São Paulo, related to the right to healthcare taking into consideration Evidence-Based Medicine.

After identifying the decisions, a qualitative analysis was made of the discussions regarding Evidence-Based Medicine. First, the decisions in which Evidence-Based Medicine was referred to but not discussed, and did not contribute to the decision. Next, the decisions in which Evidence-Based Medicine was discussed, albeit minimally. For example, a healthcare insurance plan alleges that the treatment sought is experimental, with no evidence of its efficacy, where the judge denied the argument with the assertion that there was no proof of the so-called experimental character. We sought to verify if there had been consideration of the situation of the patient/plaintiff of the action, and the efficacy or lack of efficacy of treatments or medications that are included in existing protocols to accept the measure sought legally.

It is known that it is possible that other decisions exist in which the scientific suitability of a given therapy was discussed, but that due to the fact that the complete expression “Evidence-Based Medicine” had not been included, were not located. Nevertheless, we preferred to restrict the study to the decisions that contained such a term, since adopting it as a hypothesis to be tested would be the fact of its being deemed positive by Law number 12,401/2011. That is a concern with the scientific evidence for the inclusion of new technologies by the SUS implying a growing reference to the said theoretical approach in the decisions.

It is not the role of the Judiciary to perform an in-depth analysis of Evidence-Based Medicine. This is a technical subject that requires the manifestation of experts and specialists, which is, in fact, an object of recommendation by the CNJ. The more or less adequate application of Evidence-Based Medicine also depends on the participation of the lawyers involved and on making the judges sensitive to the case. Since there were no elements for measuring such aspects, it was chosen to exam the arguments used in the decision, especially if the patient’s situation and the efficacy of the procedures available by SUS or by healthcare insurance plans were evaluated.

## RESULTS

Of the 19 decisions that referred to Evidence-Based Medicine found in the search, 6 (32%) were made in reaction to Public Authorities, thus involving the care provided by the SUS, and 13 (68%) were made relative to healthcare insurance plans – in this case, the *Associação Valeparaibana de Assistência Médica Policial* (AVAMP) and different UNIMED co-operatives.

Most of the decisions (11) were made between 2012 and 2013.^11^ One was issued in 2009,^12^ two were dated 2010,^13^ and the others, 2008^14^ (two), 2007,^15^ and 2004^16^ (also two). Therefore, an increase in frequency of identification of the presentation of arguments based on the scientific suitability of medications and procedures sought in court was observed, notably after a public hearing carried out by the Federal Supreme Court, in 2009, and the issue of recommendation 31 by the CNJ, in 2010.

The vast majority of decisions, 18 of them, were favorable to the plaintiffs. Only one decision was not favorable,^17^ in which the right to health was not discussed, but rather the right to the welfare system (a lawsuit brought against the *Instituto Nacional do Seguro Social* (INSS), seeking the concession of a benefit due to disability, *i.e.*, illness benefits).

All of the others granted what the plaintiffs intended. The treatments and procedures sought were varied, especially the number of decisions involving the provision of drug-eluting stents: four. Chart 1 summarizes the judicial decisions involving Evidence-Based Medicine between 2004 and 2013.

**Chart 1:** Court decisions involving Evidence-Based Medicine, between 2004 and 2013

Health problems considered	Number of decisions	Realm (SUS or health insurance plan)	Favorable (yes or no)
Provision of drug-eluting stents *	4	Plan	Yes
Availability of rituximab (MabThera^®^)**	1	SUS	Yes
Placement of imported orthopedic prosthesis	1		
Provision of the medication etanercept	1	SUS	Yes
Provision of Abilify^®^ to a patient affected with paranoid schizophrenia	1	SUS	Yes
Venlafaxine 150mg, Cymbalta^®^ 30mg, mirtazapine 30mg, fluoxetine 20mg, and Modafinil (STAVIGILE^®^) 200mg to treat a patient with depression**	1	SUS	Yes
Performance of percutaneous cordotomy and radiculotomy by radiofrequency	1	Plan	Yes
Performance of “dynamic spine stabilization” surgery	1	Plan	Yes
Deep bilateral cerebral electrode in patient with pain syndrome	1	Plan	Yes
Surgery with use of laser to treat urethral calculi	1	Plan	Yes
Arthrodesis in the spine of a patient affected by disk herniation	1	Plan	Yes
Placement of a tibial fixator/fastener	1	Plan	Yes
Determination to cover costs with intravitreal application of Avastin^®^ 1.25mg (bevacizumab) and of photodynamic therapy with verteporfirin to treat macular degeneration**	1	Plan	Yes
Coverage of conservative surgical procedure of tumor resection, replacing bone by homologous tissue from the bone bank, to be attached by means of an intramedullary titanium rod, and hyperbaric oxygen therapy sessions	1	Plan	Yes
Spine surgery by radiculotomy of L2 and S1 roots (with radiofrequency) ans sacral radiculotomy via the Baylis system**	1	Plan	Yes

* Two of these decisions adequately considered the situation of the plaintiff and better motivated the concession of the request: Civil Appeals 0006799-31.2010.8.26.0189 and 0044728-23.2010.8.26.0602; ** a decision that properly considered the situation of the plaintiff and better motivated granting the request. SUS: *Sistema Único de Saúde* [National Unified Healthcare System].

## DISCUSSION

A minimal discussion about Evidence-Based Medicine, or of its assumptions, that is, the suitability of the medication of procedure, as per the best scientific evidence, according to the patient’s clinical status, only occurred in ten decisions.^18^ In the others, despite being referred to, by initiative of one of the parties, Evidence-Based Medicine was not discussed, and was of no importance for the judgment, prevailing the judicial arguments bound to the content of right to healthcare, or abusive contract clauses based on the Consumer Defense Code, or on the demonstration of the experimental character of the treatment. There were cases in which it was mentioned that it should be the patient’s physician and not the health insurance plan or the State, who decides which treatment or medication should be provided.^19^ A more solidly based analysis of the peculiarity of the situation of the plaintiff and/or of the lack of value of the treatment offered by SUS, or by the healthcare insurance plan, was only made in six cases.^20^ These decisions were considered as having been more solidly based since they took into consideration the individual characteristics of each patient, since the most effective treatment for most patients is not always appropriate for a given individual, considering the possibility of hypersensitivity reactions, drug-drug interactions, and possible contraindications to certain treatments. Within this context, the role of the physician is fundamental for the right therapeutic prescription, correcting the risks of possible insufficiency of information and the generalized application of the results of clinical studies.^21^ Chart 1 summarizes the decisions analyzed. It is worrisome that in the four cases involving drug-eluting stents, it was not considered that various clinical studies concluded that there were no significant differences among them in terms of the events of death, thrombosis, infarct, and need for reoperation. The two most recent decisions involving stents highlighted in chart 1, at least presented as rational the peculiarities of the plaintiffs, such as their age and the diseases they suffer from.

## CONCLUSION

Despite being referred to, Evidence-Based Medicine was not used as a basis for most of the decisions, nor did it contribute towards a more adequate analysis of the patient’s situation, with the prevalence of judicial arguments linked to the superiority of the right to healthcare, based on Article 196 of the Constitution, and to the abusive and illegal character of the restrictions to the provision of drugs and treatments, based on the Consumer Defense Code. The number of decisions in which most consideration was given to scientific evidence and to the peculiarities of the patients is troublesome. It should be reminded that its lack of consideration leads to the supply of unnecessary or inadequate medications and treatments, ignoring alternatives provided by health insurance plans and by the *Sistema Único de Saúde*, increasing the burden of the public health system and insurance plans. It is necessary that the National Court Council evaluate its recommendation 31 that, even without having a binding character, establishes obvious premises to avoid decisions that are not based on the best scientific knowledge available. It is necessary to broaden the discussion of Evidence-Based Medicine in processes involving public health, since it represents an extremely useful tool in helping judicial decision. Nevertheless, its inadequate and insufficient application, as was perceived in this study, points to the need to prepare the members of the Judiciary, of the Public Prosecutor’s Office, and of public and private law firms, as to its use, which can contribute to better founded decisions and to a better quality in the resulting expenditures.
