# Supplementary material for: Evidence-Based Medicine in judicial decisions concerning right to healthcare
Source: Einstein (Sao Paulo). 2016 Jan-Mar;14(1):1–5. doi: 10.1590/S1679-45082016AO3363 (PMC4872909; doi:10.1590/S1679-45082016AO3363)
Supplement: Translation

# Translation: A Medicina Baseada em Evidências na jurisprudência relativa ao direito à saúde

**Authors:** Eduardo Rocha Dias, Geraldo Bezerra da Silva

**Affiliations:** 1 Programa de Pós-Graduação em Direito Constitucional, Universidade de Fortaleza, Fortaleza, CE, Brasil.; 2 Programa de Pós-Graduação em Saúde Coletiva, Universidade de Fortaleza, Fortaleza, CE, Brasil.

**Keywords:** Medicina Baseada em Evidências, Direito à Saúde, Saúde/legislação & jurisprudência

## Abstract

**Objetivo:**

Analisar, a partir do exame de decisões proferidas por tribunais brasileiros, como a Medicina Baseada em Evidências foi aplicada e se conduziu a decisões bem fundamentadas, sob a perspectiva do melhor conhecimento científico.

**Métodos:**

Analisaram-se decisões de Tribunais Federais selecionadas pela busca, sem limitação temporal, no sítio do Conselho da Justiça Federal, dedicado à pesquisa unificada de jurisprudência, contendo a expressão “Medicina Baseada em Evidências”. Quanto aos acórdãos da Corte Paulista, a busca foi feita em sua página na internet dedicada à pesquisa de jurisprudência, também por meio da expressão “Medicina Baseada em Evidências”, e sem limitação temporal. Efetuou-se, a seguir, uma análise qualitativa da discussão efetuada em cada processo, verificando se nela se apreciaram a situação do paciente/autor da ação, e a eficácia ou ineficácia de tratamentos ou medicamentos constantes em protocolos já existentes, antes de se deferir a medida buscada em juízo.

**Resultados:**

Verificou-se que em menos de um terço das decisões examinadas houve uma discussão adequada da eficácia do procedimento buscado judicialmente, em comparação com os disponibilizados em protocolos clínicos pelo Sistema Único de Saúde e por planos privados de saúde, à luz da situação individual do autor da ação. A maioria das decisões envolvia planos de saúde (n=13, 68%).

**Conclusão:**

O número de decisões em que se deu maior consideração à evidência científica e às peculiaridades dos pacientes foi preocupante. É necessário ampliar a discussão da Medicina Baseada em Evidências nos processos envolvendo a saúde pública.

## INTRODUÇÃO

Muito se discute sobre a judicialização do acesso a cuidados de saúde no Brasil. O elevado grau de interferência judicial na determinação do fornecimento de medicamentos e de outras prestações de saúde afeta os orçamentos públicos e a equidade no atendimento dos seus beneficiários.^1^ Também no âmbito dos planos e seguros de saúde, a intervenção judicial tem sido crescentemente buscada por consumidores pretendendo efetivar seus direitos.^2^ Além da doutrina,^3^ o próprio Judiciário tem buscado encaminhar a discussão do problema, como, por exemplo, na audiência pública realizada entre abril e maio de 2009 pelo Supremo Tribunal Federal.^4^

O Conselho Nacional de Justiça (CNJ), órgão de controle da atuação administrativa e financeira do Poder Judiciário, editou a recomendação 31, de 30 de março de 2010,^5^ exortando Tribunais Federais e Estaduais a celebrarem convênios que objetivem disponibilizar apoio técnico por médicos e farmacêuticos para auxiliar os magistrados na formação de um juízo de valor quanto à apreciação das questões clínicas nas ações relativas à saúde. Também se pediu aos tribunais que orientassem os magistrados a evitar autorizar o fornecimento de medicamentos ainda não registrados pela Agência Nacional de Vigilância Sanitária (ANVISA) ou experimentais.

Houve, ainda, a instituição, pelo CNJ, do Fórum Nacional do Judiciário, para monitoramento e resolução das demandas de assistência à saúde – Fórum da Saúde, por meio da sua resolução 107, de 6 de abril de 2010,^6^ cuja área de atuação passou a incluir posteriormente a saúde suplementar.

Verifica-se, assim, que o tema da judicialização da saúde recomenda a adoção de soluções guiadas pela melhor técnica, o que, infelizmente, nem sempre ocorre. Para orientar a decisão judicial sobre fornecimento de determinado tratamento buscado em juízo, a abordagem conhecida como Medicina Baseada em Evidências parece oferecer contribuições interessantes. Não por acaso, a busca do melhor conhecimento científico, na incorporação de novas tecnologias, foi acolhida pela legislação brasileira em decorrência da lei 12.401, de 28 de abril de 2011, que alterou a lei 8.080/1990, nela inserindo um Capítulo VIII no seu Título II. Conforme previsão do art. 19-Q da lei 8.080/1990, acrescentado pela lei 12.401/2011, na incorporação, na exclusão ou na alteração pelo Sistema Único de Saúde (SUS) de novos medicamentos, produtos e procedimentos, bem como na constituição ou na alteração de protocolo clínico ou de diretriz terapêutica, devem ser consideradas as evidências científicas sobre a eficácia, a acurácia, a efetividade e a segurança do medicamento, produto ou procedimento objeto do processo.

O art. 19-T da lei 8.080/1990, por sua vez, veda o pagamento, o ressarcimento ou o reembolso de medicamento, produto e procedimento clínico ou cirúrgico experimental, ou de uso não autorizado pela ANVISA, bem como a dispensação, o pagamento, o ressarcimento ou o reembolso de medicamento e produto, nacional ou importado, sem registro na mencionada agência.

Desse modo, observa-se que são buscados dois tipos de soluções em relação à judicialização: uma interna ao Poder Judiciário, por meio da audiência pública acima referida e da atuação do CNJ, que não vincula os juízes, mas que estabelece diretrizes as quais podem levar a decisões melhor fundamentadas; outra, por meio do próprio Executivo, pela lei 12.401/2011, ao prever critérios científicos para a incorporação de novas tecnologias, o que passa também a constituir matéria que o Judiciário, ao apreciar os pedidos das partes, terá que analisar.^7^

Cumpre verificar como o Judiciário tem atuado no tocante à exigência de eficácia científica dos procedimentos e medicamentos. Ao determinar o fornecimento de um medicamento ou de um tratamento, ele tem considerado os protocolos clínicos existentes? Para afastar a aplicação dos protocolos, as decisões têm apreciado as peculiaridades dos pacientes e a ineficácia dos tratamentos disponibilizados pelo SUS ou por planos de saúde privados? As recomendações do CNJ têm sido seguidas?

## OBJETIVO

Analisar, a partir do exame de decisões proferidas por tribunais brasileiros, como a Medicina Baseada em Evidências foi aplicada e se conduziu a decisões bem fundamentadas, sob a perspectiva do melhor conhecimento científico.

## MÉTODOS

O presente estudo propõe-se a apresentar uma análise crítica de decisões de tribunais brasileiros, sendo três decisões proferidas por Tribunais Regionais Federais e 16 decisões do Tribunal de Justiça do Estado de São Paulo relacionadas ao direito à saúde, levando em consideração a Medicina Baseada em Evidências.

A escolha das decisões dos Tribunais Federais decorreu de busca, sem limitação temporal, no sítio do Conselho da Justiça Federal, dedicado à pesquisa unificada de jurisprudência.^8^ Trata-se de sítio em que se tem acesso a mecanismo de busca de decisões proferidas pelo Supremo Tribunal Federal e pelo Superior Tribunal de Justiça, cortes de maior hierarquia no Brasil e responsáveis pela uniformização da interpretação da Constituição e das leis, respectivamente. Nesse sítio, também se podem pesquisar as decisões exaradas pelos cinco Tribunais Regionais Federais, que apreciam recursos de decisões em que a União e entes federais são interessados, pela Turma Nacional de Uniformização de Jurisprudência (TNU), pelas Turmas Regionais de Uniformização e pelas Turmas Recursais dos Estados da Federação, que também apreciam recursos de decisões proferidas em ações que seguem a lei dos Juizados Especiais Federais, de interesse da União e entes federais. A pesquisa pode ser feita utilizando-se termos específicos, separados ou em conjunto, pelo emprego de modais como “e”, “ou”, “adj” e outros. Procuraram-se decisões proferidas por referidos órgãos do Poder Judiciário que contivessem a expressão “Medicina Baseada em Evidências” (entre aspas). Assim, somente foram apontadas decisões em que a expressão completa foi mencionada. As ações judiciais envolvendo o direito à saúde podem envolver todos os entes da Federação (União, Estados, Municípios e Distrito Federal). Quando a União é parte, a competência para tais ações é da Justiça Federal. Trata-se de decisões envolvendo, então, o SUS. Foram encontrados apenas três acórdãos (decisões de órgãos colegiados de tribunais), dos Tribunais Regionais Federais da 2ª, 5ª e 4ª Regiões, com sede no Rio de Janeiro (RJ), Recife (PE) e Porto Alegre (RS), respectivamente.

Quanto aos acórdãos da Corte Paulista, a busca foi feita na sua página na internet, na parte dedicada à pesquisa de jurisprudência,^9^ também por meio da expressão “Medicina Baseada em Evidências”, e sem limitação temporal. À semelhança do sítio do Conselho da Justiça Federal, a pesquisa pode ser feita por termos isolados ou mencionados em conjunto nas decisões. Também se pesquisou a expressão completa entre aspas, o que permitiu localizar as decisões em que ela foi mencionada. Como se trata de Tribunal de Justiça estadual, a União não é parte dessas ações, que são movidas contra entes privados (planos e seguros de saúde) e contra o Estado de São Paulo e/ou Municípios daquela unidade da Federação, dizendo respeito, no segundo caso, ao SUS.

A pesquisa foi feita no sítio do Tribunal de Justiça do Estado de São Paulo, por este ser o mais populoso e com maior grau de cobertura dos planos e seguros privados de saúde, conforme dados da Agência Nacional de Saúde Suplementar.^10^

Foram identificadas 19 de decisões de tribunais brasileiros, sendo 3 decisões proferidas por Tribunais Regionais Federais e 16 decisões do Tribunal de Justiça do Estado de São Paulo, relacionadas ao direito à saúde levando em consideração a Medicina Baseada em Evidências.

Após a identificação das decisões, foi feita uma análise qualitativa da discussão nela contida sobre a Medicina Baseada em Evidências. Em primeiro lugar, identificaram-se as decisões em que ela, apesar de referida, não foi discutida, nem contribuiu para a decisão. Em seguida, destacaram-se as decisões em que houve uma discussão da Medicina Baseada em Evidências, ainda que mínima. Por exemplo, a alegação de um plano de saúde de que o tratamento buscado é experimental, não havendo evidências de sua eficácia, tendo o julgador afastado tal argumento com a afirmação de que não houve prova de tal caráter experimental. Buscou-se verificar se foram apreciadas a situação do paciente/autor da ação, e a eficácia ou ineficácia de tratamentos ou medicamentos constantes em protocolos já existentes para deferir a medida buscada em juízo.

Não se ignora que é possível que existam outras decisões em que se tenha discutido a adequação científica de uma dada terapia, mas que, por não incorporarem a expressão completa “Medicina Baseada em Evidências”, não tenham sido localizadas. No entanto, preferiu-se restringir a pesquisa às decisões que contenham tal termo diante do fato de se adotar como hipótese a ser testada a de que a positivação pela lei 12.401/2011 de uma preocupação com as evidências científicas para a incorporação de novas tecnologias pelo SUS implica uma crescente referência à aludida abordagem teórica nas decisões.

Não é papel do Judiciário realizar uma análise aprofundada da Medicina Baseada em Evidências. Trata-se de matéria técnica, que exige manifestação de peritos e especialistas, o que é, inclusive, objeto de recomendação pelo CNJ. A aplicação mais ou menos adequada da Medicina Baseada em Evidências depende também da atuação dos advogados envolvidos e da sensibilização dos magistrados. Como não existiam elementos para aferir tais aspectos, privilegiou-se o exame dos argumentos utilizados na decisão, em especial se foram apreciadas situação do paciente e da eficácia dos procedimentos disponibilizados pelo SUS ou por planos de saúde.

## RESULTADOS

Das 19 decisões que referiram a Medicina Baseada em Evidências localizadas na pesquisa, 6 (32%) foram proferidas em face do Poder Público, envolvendo, desse modo, o atendimento pelo SUS, e 13 (68%) foram proferidas face a planos de saúde − no caso a Associação Valeparaibana de Assistência Médica Policial (AVAMP) e diferentes cooperativas da UNIMED.

A maioria das decisões (11) foi proferida entre 2012 e 2013.^11^ Uma foi proferida em 2009,^12^ duas datavam de 2010^13^ e as restantes, de 2008^14^ (duas), 2007^15^ e 2004^16^ (também duas). Verificou-se, assim, um incremento na frequência com que se identificou a apresentação de argumentos fundados na adequação científica de medicamentos e procedimentos buscados em juízo, notadamente após a audiência pública realizada pelo Superior Tribunal Federal, em 2009, e a edição da recomendação 31 pelo CNJ, em 2010.

A esmagadora maioria das decisões foi favorável aos autores: 18 delas. Apenas uma decisão foi desfavorável,^17^ na qual não se discutiu o direito à saúde, mas sim à previdência social (ação movida contra o Instituto Nacional do Seguro Social − INSS, visando à concessão de um benefício por incapacidade, o auxílio-doença).

Todas as demais concederam o que os autores pretendiam. Os tratamentos e procedimentos buscados foram variados, sendo digno de nota o número de decisões envolvendo o fornecimento de *stents* revestidos de medicamentos: quatro. O quadro 1 resume as decisões judiciais envolvendo a Medicina Baseada em Evidências entre 2004 e 2013.

**Quadro 1:** Decisões judiciais envolvendo a Medicina Baseada em Evidências entre 2004 e 2013

Problema de saúde em questão	Número de decisões	Âmbito (SUS ou plano de saúde)	Favorável (sim ou não)
Fornecimento de *stents* revestidos de medicamentos*	4	Plano	Sim
Disponibilização de rituximabe (MabThera^®^)**	1	SUS	Sim
Implantação de prótese ortopédica importada	1		
Fornecimento do medicamento etanercepte	1	SUS	Sim
Fornecimento de Abilify^®^ a paciente acometida de esquizofrenia paranoide	1	SUS	Sim
Venlafaxina 150mg, Cymbalta^®^ 30mg, mirtazapina 30mg, fluoxetina 20mg e Modafinil (STAVIGILE^®^) 200mg para tratamento de paciente com depressão**	1	SUS	Sim
Realização de cordotomia e radiculotomia percutânea por radiofrequência	1	Plano	Sim
Realização de cirurgia de “estabilização dinâmica da coluna”	1	Plano	Sim
Eletrodo cerebral profundo bilateral em paciente acometida de síndrome álgica	1	Plano	Sim
Cirurgia com emprego de laser para tratamento de cálculo no ureter	1	Plano	Sim
Cirurgia de artrodese de coluna em paciente acometida de hérnia de disco	1	Plano	Sim
Colocação de fixador na tíbia	1	Plano	Sim
Determinação para cobrir custos com a aplicação intravítrea de Avastin^®^ 1,25mg (bevacizumabe) e com terapia fotodinâmica com verteporfirina para tratar degeneração macular**	1	Plano	Sim
Cobertura de procedimento cirúrgico conservador de ressecção de lesão tumoral, com substituição de osso por homólogo de banco de ossos a ser fixado por haste intramedular de titânio, e de sessões de oxigenoterapia hiperbárica	1	Plano	Sim
Cirurgia de coluna por meio de radiculotomia de raiz L2 e S1 (com radiofrequência) e racidulotomia sacral pelo sistema Baylis**	1	Plano	Sim

* Duas dessas decisões consideraram adequadamente a situação do autor e motivaram melhor a concessão de seu pedido: Apelações Cíveis 0006799-31.2010.8.26.0189 e 0044728-23.2010.8.26.0602; ** trata-se de decisão que considerou adequadamente a situação do autor e motivou melhor a concessão do pedido. SUS: Sistema Único de Saúde.

## DISCUSSÃO

Uma discussão mínima da Medicina Baseada em Evidências, ou de seus pressupostos, ou seja, a adequação do medicamento ou procedimento, conforme as melhores evidências científicas, de acordo com o quadro do paciente, ocorreu em apenas dez decisões.^18^ Nas demais, apesar de referida, por iniciativa de alguma das partes, a Medicina Baseada em Evidência não foi discutida, não tendo importância para o julgamento, e prevalecendo argumentos jurídicos ligados ao conteúdo do direito à saúde ou à abusividade de cláusulas contratuais com base no Código de Defesa do Consumidor, ou à não demonstração do caráter experimental do tratamento. Houve casos em que se referiu que deve ser o médico do paciente, e não o plano de saúde ou o Estado, quem deve decidir qual tratamento ou medicamento deve ser fornecido.^19^ Uma análise mais fundamentada da peculiaridade da situação do autor e/ou da imprestabilidade do tratamento disponibilizado pelo SUS ou pelo plano de saúde somente foi feita em seis casos.^20^ Consideram-se melhor fundamentadas essas decisões por terem apreciado as características individuais de cada paciente, pois nem sempre o tratamento mais eficaz para a maioria dos pacientes é adequado ao indivíduo em questão, tendo-se em vista a possibilidade de reações de hipersensibilidade, interações medicamentosas e possíveis contraindicações a determinados tratamentos. Nesse contexto, o papel do médico é fundamental para a correta prescrição terapêutica, corrigindo os riscos de eventual insuficiência da informação disponível e da aplicação generalizada dos resultados dos estudos clínicos.^21^ O quadro 1 resume as decisões analisadas. Mostra-se preocupante que, nos quatro casos envolvendo o fornecimento de *stents* revestidos de medicamentos, cinco vezes mais caros que os não revestidos, tenha se desconsiderado que vários estudos clínicos concluíram não haver diferenças significativas entre eles em termos dos eventos morte, trombose, infarto e necessidade de nova cirurgia. As duas decisões mais recentes envolvendo *stents*, destacadas no quadro 1, ao menos apresentaram como justificativas peculiaridades dos autores, como sua idade e doenças que os acometem.

## CONCLUSÃO

Apesar de referida, a Medicina Baseada em Evidências não foi utilizada como fundamento da maioria das decisões, nem contribuiu para uma análise mais adequada da situação do paciente, prevalecendo argumentos jurídicos ligados à superioridade do direito à saúde, com fundamento no art. 196 da Constituição, e ao caráter abusivo e ilegal de restrições ao fornecimento de medicamentos e tratamentos, com base no Código de Defesa do Consumidor. O número de decisões em que se deu maior consideração à evidência científica e às peculiaridades dos pacientes é preocupante. Deve ser lembrado que sua desconsideração leva ao fornecimento de medicamentos e tratamentos desnecessários ou inadequados, ignorando alternativas disponibilizadas por planos e saúde e pelo Sistema Único de Saúde, onerando o sistema público de saúde e os planos. É necessário que o Conselho Nacional de Justiça avalie o atendimento à sua recomendação 31 que, mesmo sem ter caráter vinculante, estabelece premissas óbvias para evitar decisões que não tenham embasamento no melhor conhecimento científico disponível. É necessário ampliar a discussão da Medicina Baseada em Evidências nos processos envolvendo a saúde pública, pois ela representa uma ferramenta extremamente útil para auxílio em decisões judiciais. No entanto, sua inadequada e insuficiente aplicação, conforme constatado na pesquisa, aponta para a necessidade de capacitar os membros do Judiciário, do Ministério Público e da Advocacia, pública e privada, quanto à sua utilização, o que pode contribuir para decisões melhor fundamentadas e para uma maior qualidade do gasto delas decorrente.
